# Supplementary material for: A Novel Therapeutic Formulation for the Improved Treatment of Indian Red Scorpion (Mesobuthus tamulus) Venom-Induced Toxicity-Tested in Caenorhabditis elegans and Rodent Models
Source: Toxins (Basel). 2023 Aug 14;15(8):504. doi: 10.3390/toxins15080504 (PMC10467153; doi:10.3390/toxins15080504)
Supplement: Supplementary file 1 [file toxins-15-00504-s001.zip › toxins-2460079-supplementary.pdf]

**Supporting information**

| Sl. No. | Name | Brief caption |
|---|---|---|
| **1** | **Supplementary Table S1** | Composition for the components |
| **2** | **Supplementary Table S2** | Docking scores of the α1 adrenoreceptor antagonist (AAA) with the α1 adrenergic receptors from humans, mice, and *C. elegans*. BA: Binding affinity; DR: Docking rank. |
| **3** | **Supplementary Table S3** | Determination of toxicity of ASA, AAA and Ascorbic acidin *C. elegans* |
| **4** | **Supplementary Table S4** | Determination of *in vivo* optimum dose of commercial ASA, Ascorbic acid, and AAA against $LC_{50}$ value of MTV. |
| **5** | **Supplementary Table S5** | Physical or behavioral changes in MTV-treated albino Wistar strain rat and their recovery by formulated drug 2 [ASA (187.5 µg), Ascorbic acid (0.1 µg), AAA (3 µM)]. The symptoms were noted 24 h post-injection of MTV. |
| **6** | **Supplementary Figure S1** | Homology modelled structures of the following proteins taken from SwissProt [Structure through AlphaFold]. Protein-ligand interactions of α1 adrenoreceptor antagonist (AAA) with α1 adrenergic receptor. |
| **7** | **Supplementary Figure S2** | The $LC_{50}$ value of *C. elegans* N2 calculated after 24h treatment of scorpion venom (*M. tamulus* venom). The $LC_{50}$ value calculated for Scorpion venom towards *C. elegans*, after 24 h incubation, was 125 µg/ml. |

| | | |
|---|---|---|
| **8** | **Supplementary Figure S3** | Fluorescence image of confocal microscopy of *M. tamulus* venom induced ROS generation in *C. elegans* after 6h of *M. tamulus* venom ($LC_{50}$ concentration) treatment and its neutralization by Prazosin, Silodosin and Terazosin. ROS level in positive control (CCCP1) *C. elegans* was considered as baseline (100%) and other values were compared with that. |
| **9** | **Supplementary Figure S4** | Fluorescence image of confocal microscopy of MTV-induced alteration of mitochondrial membrane potential and its neutralization by Prazosin, Silodosin and Terazosin. ROS level in positive control (CCCP1) *C. elegans* was considered as baseline (100%) and other values were compared with that. |
| **10** | **Supplementary Figure S5** | shows the DPPH free radical-scavenging activity of **(a)** the optimum dose of individual formulation components, their combinations, and different concentrations of formulations, **(b)** Individual components of the formulation and their combinations compared with formulation 2. Data represent mean ± SD of three determinations. Significance of difference, *$p\leq0.05$ as compared to formulation 2. There was no significant difference ($p>0.05$) between formulations 2 and 3. |
| **11** | **Supplementary Figure S6** | Histopathological analysis of the *M. tamulus* venom-induced Wistar rat tissues and their neutralisation by formulation 2. Light microscopic observation of a) Heart, b) Kidney, c) Liver, d) Lung, e) Testis and f) Ovary from control and treated groups, Bar-100µM. The black arrow indicates the morphological |

| | | |
|---|---|---|
| | | changes observed in MTV-induced rat tissue compared to the control |
| **12** | **Supplementary Figure S7** | Circulating levels of pro-inflammatory cytokines in the Swiss albino mice with and without *Mesobuthus tamulus* envenomation, MTV-treated plasma and control plasma, respectively. Significance of difference, *$p \leq 0.05$ as compared to control |

**Supplementary Table S1.** Composition of the components used for formulation

| **S. No.** | **Component of the formulation** | **Solubility** |
|---|---|---|
| **1** | ASA (% venom specific antibodies: 6.29%) | Water/ 1xPBS |
| **2** | AAA (Prazosin-HCL) | Water/ 1XPBS and then heating at 50-60 °C for 3-5 min |
| **3** | Ascorbic acid | Water/ 1XPBS |

**Supplementary Table S2.** Docking scores of the α1 adrenoreceptor antagonist (AAA) with the α1 adrenergic receptors from humans, mice, and *C. elegans*. BA: Binding affinity; DR: Docking rank.

| **Receptor** | **Docking score with prazosin hydrochloride [PMCID: 68546]** | | | | **Docking score with terazosin hydrochloride [PMCID: 68546]** | | | | **Docking score with silodosin [PMCID: 68546]** | | | |
|---|---|---|---|---|---|---|---|---|---|---|---|---|
| | **BA** | **rmsd/ub** | **rmsd/lb** | **DR** | **BA** | **rmsd/ub** | **rmsd/lb** | **DR** | **BA** | **rmsd/ub** | **rmsd/lb** | **DR** |
| Alpha1A adrenergic receptor, (AAAR) (human) | -7.7 | 4.372 | 2.808 | 1 | -6.8 | 27.117 | 24.472 | 5 | -6.9 | 2.866 | 1.929 | 4 |
| Alpha1B adrenergic receptor (ABAR) (human) | -6.4 | 30.045 | 26.824 | 7 | -7.1 | 17.451 | 13.724 | 4 | -7 | 33.115 | 29.702 | 3 |
| Alpha1D adrenergic receptor (ADAR) (human) | -6.9 | 44.121 | 40.803 | 4 | -6.5 | 41.723 | 37.361 | 6 | -7.6 | 2.399 | 1.596 | 2 |
| Alpha1A adrenergic receptor (AAAR) (mouse) | -6.7 | 18.454 | 16.899 | 5 | -6.5 | 19.056 | 16.835 | 6 | -6.7 | 60.691 | 57.074 | 6 |
| Alpha1B adrenergic receptor (ABAR) (mouse) | -7.1 | 2.835 | 1.907 | 3 | -7.3 | 17.909 | 17.004 | 3 | -6.4 | 23.825 | 19.373 | 7 |
| Alpha1D adrenergic receptor (ADAR) (mouse) | -7.3 | 27.734 | 25.635 | 2 | -7.6 | 18.07 | 16.309 | 1 | -7.7 | 37.322 | 31.764 | 1 |
| SER6 receptor (*C. elegans*) | -6.6 | 8.822 | 4.309 | 6 | -7.5 | 15.602 | 10.429 | 2 | -6.8 | 43.083 | 40.303 | 5 |

**Supplementary Table S3.** Determination of toxicity of ASA, AAA and Ascorbic acid in *C. elegans.* Data represent mean ± SD of three determinations.

| **S. No.** | **Components** | **Viability of *C. elegans* (%)** | |
|---|---|---|---|
| | | **0 h** | **24 h** |
| 1 | Control | 100.00 ± 5 | 97.20 ± 4.86 |
| 2 | ASA (PSVPL) | 100.00 ± 5 | 96.10 ± 4.81 |
| 3 | ASA (HBC) | 100.00 ± 5 | 95.92 ± 4.73 |
| 4 | AAA (Prazosin) | 100.00 ± 5 | 96.78 ± 4.80 |
| 5 | AAA (Silodosin) | 100.00 ± 5 | 97.13 ± 4.69 |
| 6 | AAA (Terazosin) | 100.00 ± 5 | 96.98 ± 4.84 |

**Supplementary Table S4.** Determination of *in vivo* optimum dose of commercial ASA, Ascorbic acid, and AAAs against $LC_{50}$ of *M. tamulus* venom.

| S. No. | Name of samples | Optimum dose |
|---|---|---|
| **1** | ASA (PSVPL and HBC) | 1500 µg |
| **2** | Ascorbic acid | 1 µg |
| **3** | AAAs:<br>Prazosin,<br>Terazosin<br>Silodosin | <br>50 µM<br>50 µM<br>25 µM |

**Supplementary Table S5.** Physical or behavioral changes in MTV-treated albino Wistar strain rat and their recovery by formulation 2 [ASA (187.5 µg), Ascorbic acid (0.1 µg), AAA (3 µM)]. The symptoms were noted 24 h post-injection of MTV.

| S. No. | Pathophysiological symptoms post MTV-treatment | Treatment with the formulation 2 |
|---|---|---|
| **1** | High urination | Recovered |
| **2** | Fast breathing | Recovered |
| **3** | Defecation | Recovered |
| **4** | Become more thirsty | Recovered |
| **5** | Weak grip strength | Recovered |

## Protein-ligand interactions:

**Generation of the 3D structure of Prazosin Hydrochloride [PMCID: 68546]:**

**Canonical SMILES:**
COC1=C(C=C2C(=C1)C(=NC(=N2)N3CCN(CC3)C(=O)C4=CC=CO4)N)OC.Cl

**Molecular Formula:**

$C_{19}H_{22}ClN_5O_4$

3D structure was generated using Corina Classic software.

**Generation of the 3D structure of Terazosin hydrochloride [PMCID: 44383]:**

**Canonical SMILES:**
COC1=C(C=C2C(=C1)C(=NC(=N2)N3CCN(CC3)C(=O)C4CCCO4)N)OC.Cl

**Molecular Formula:**

$C_{19}H_{26}ClN_5O_4$

3D structure was generated using Corina Classic software.

**Generation of the 3D structure of Silodosin [PMCID: 5312125]:**

**Canonical SMILES:**
CC(CC1=CC2=C(C(=C1)C(=O)N)N(CC2)CCCO)NCCOC3=CC=CC=C3OCC(F)(F)F

**Molecular Formula:**

$C_{25}H_{32}F_3N_3O_4$

3D structure was generated using Corina Classic software.

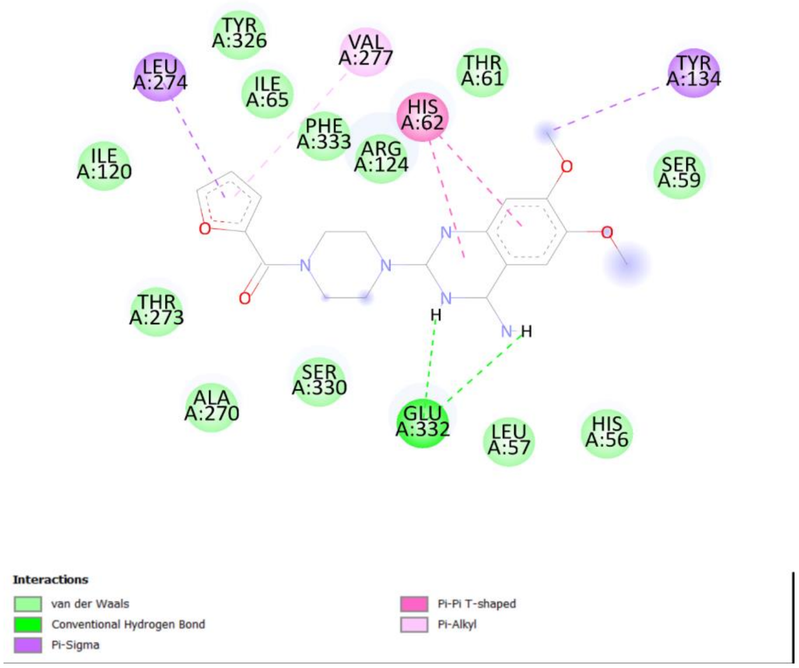


**1.a AAAR (human) + Prazosin-HCL** [AlphaFold identifier of AAAR: AF-P35348-F1, SwissProt Accession No.: P35348]

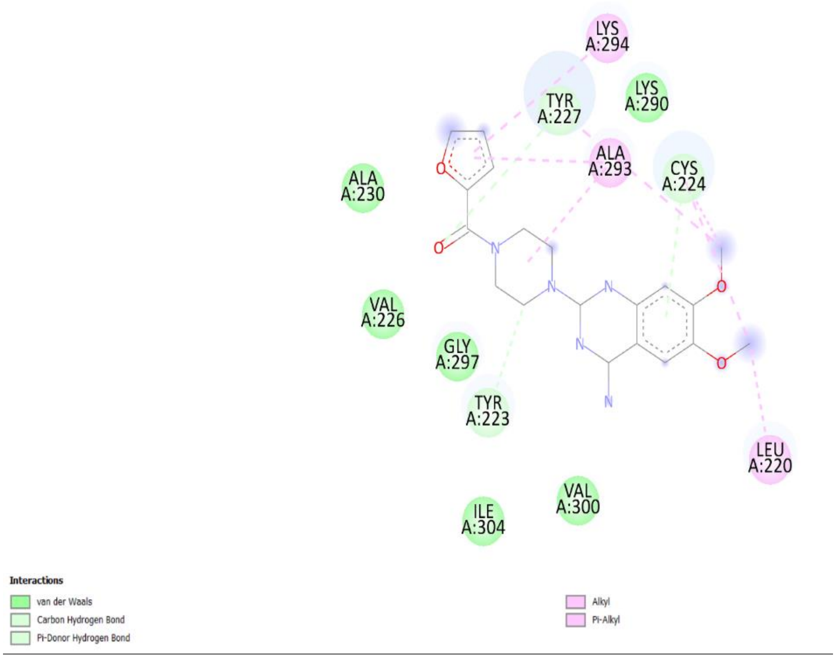


**1.b ABAR (human)+ Prazosin-HCL** [AlphaFold identifier of ABAR: AF-P35368-F1, SwissProt Accession No.: P35368]

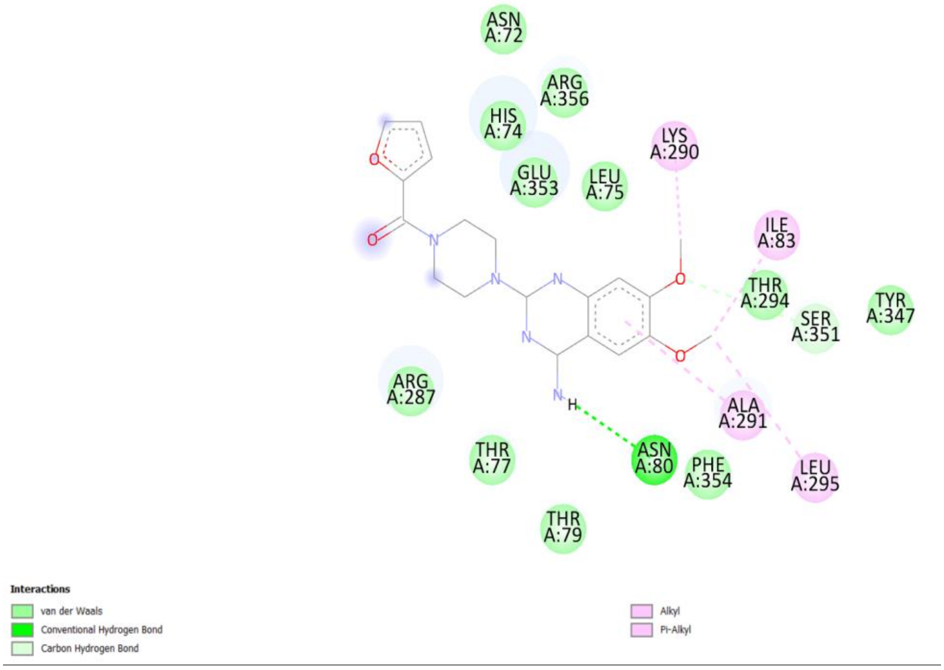


**1.c ADAR (human)+ Prazosin-HCL** [AlphaFold identifier of ADAR: AF-P25100-F1, SwissProt Accession No.: P25100]

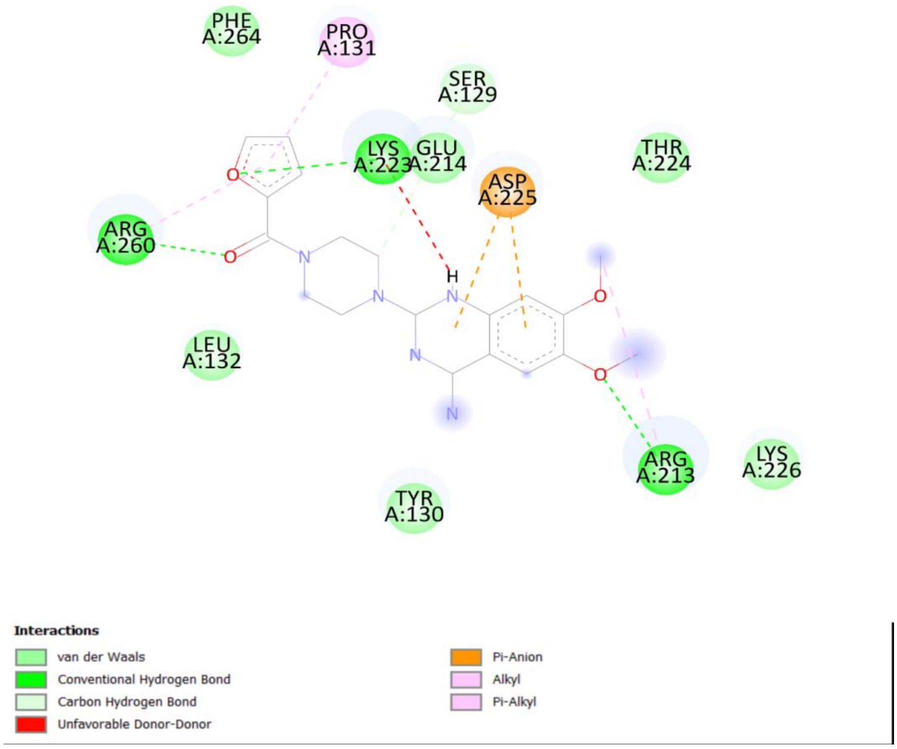


**1.d AAAR (Mouse)+ Prazosin-HCL** [AlphaFold identifier of AAAR: AF-P97718-F1, SwissProt Accession No.: P97718]

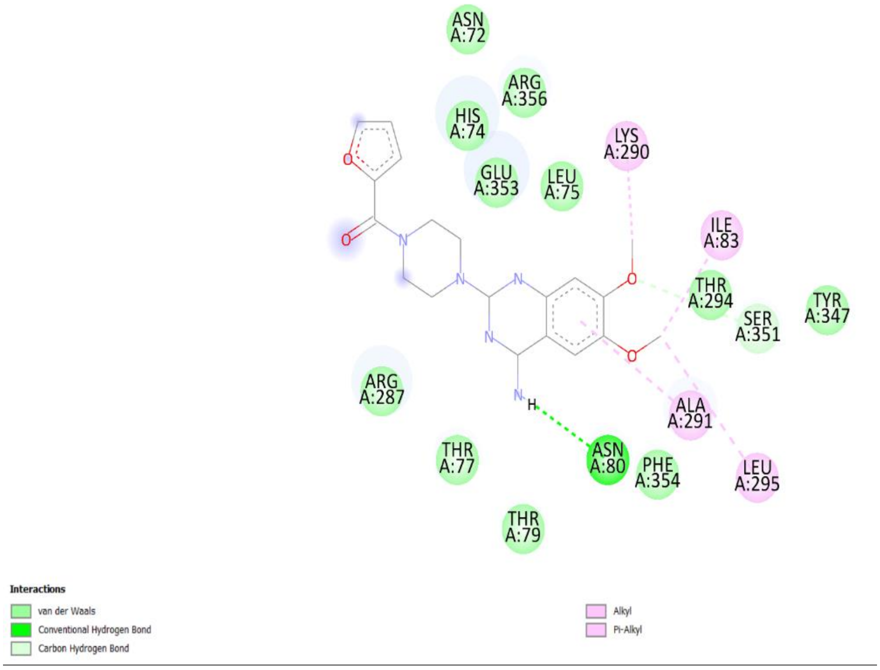


**1.e ABAR (Mouse)+ Prazosin-HCL** [AlphaFold identifier of ABAR: AF-P97717-F1, SwissProt Accession No.: P97717]

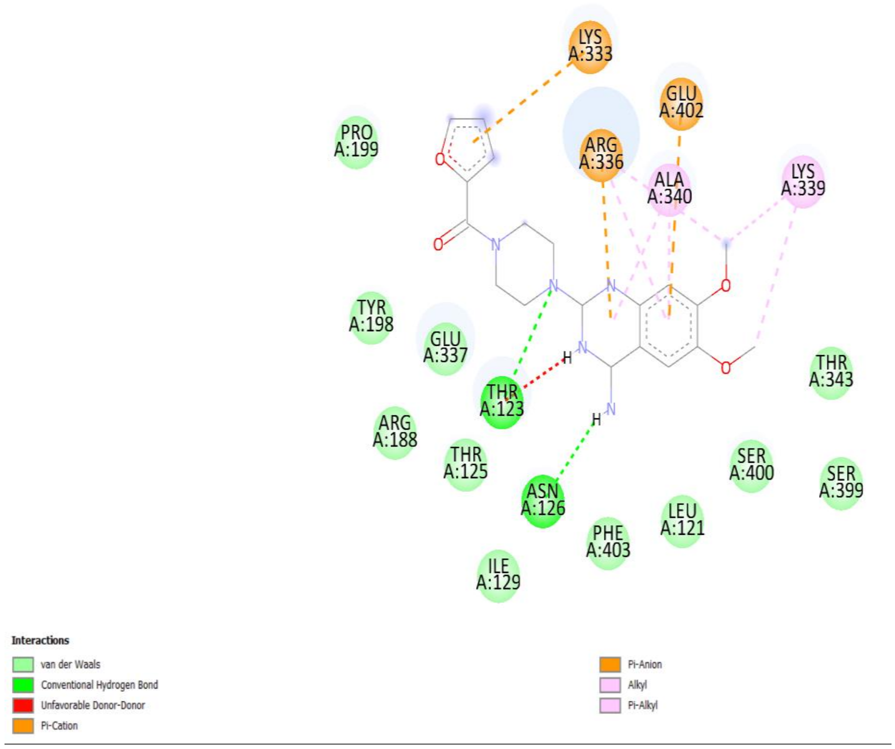


**1.f ADAR (Mouse)+ Prazosin-HCL** [AlphaFold identifier of ADAR: AF-P97714-F1, SwissProt Accession No.: P97714]

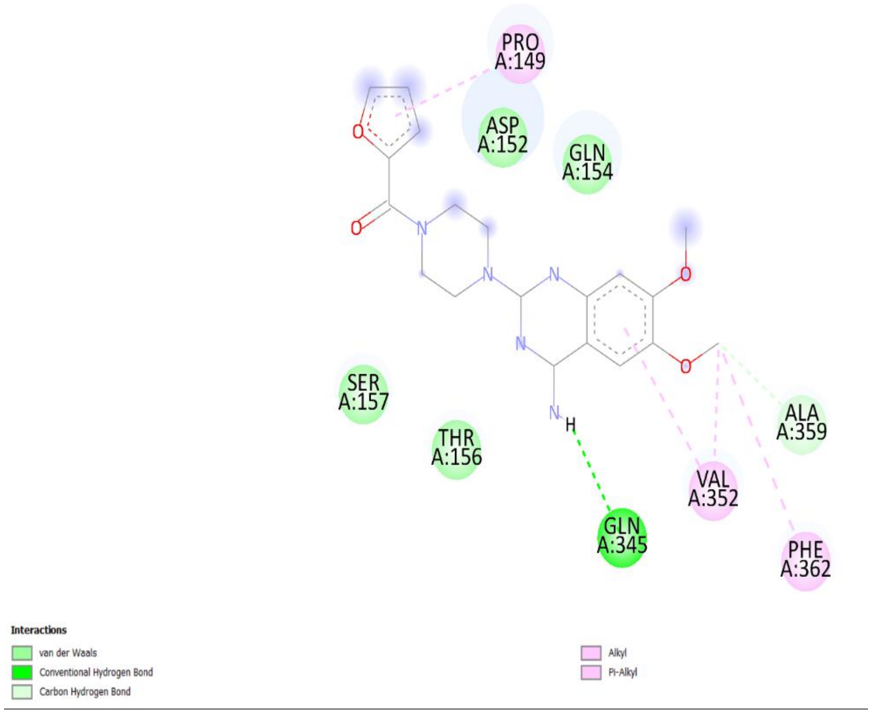


**1.g SER6 receptor (*C. elegans*)+ Prazosin-HCL** [AlphaFold identifier: AF-Q8MXS7-F1, SwissProt Accession No.: Q8MXS7]

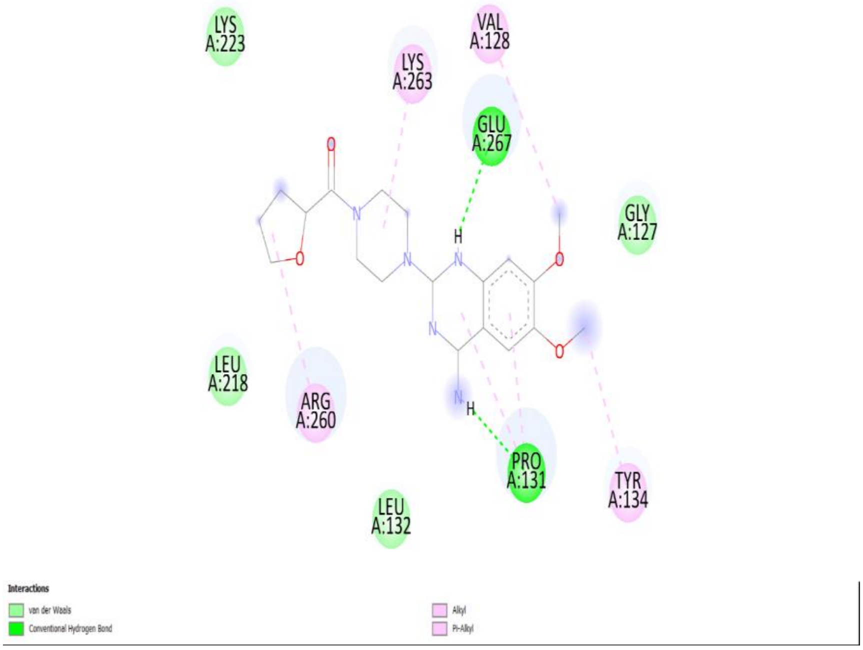


**1.h AAAR (human) + Terazosin-HCL** [AlphaFold identifier of AAAR: AF-P35348-F1, SwissProt Accession No.: P35348]

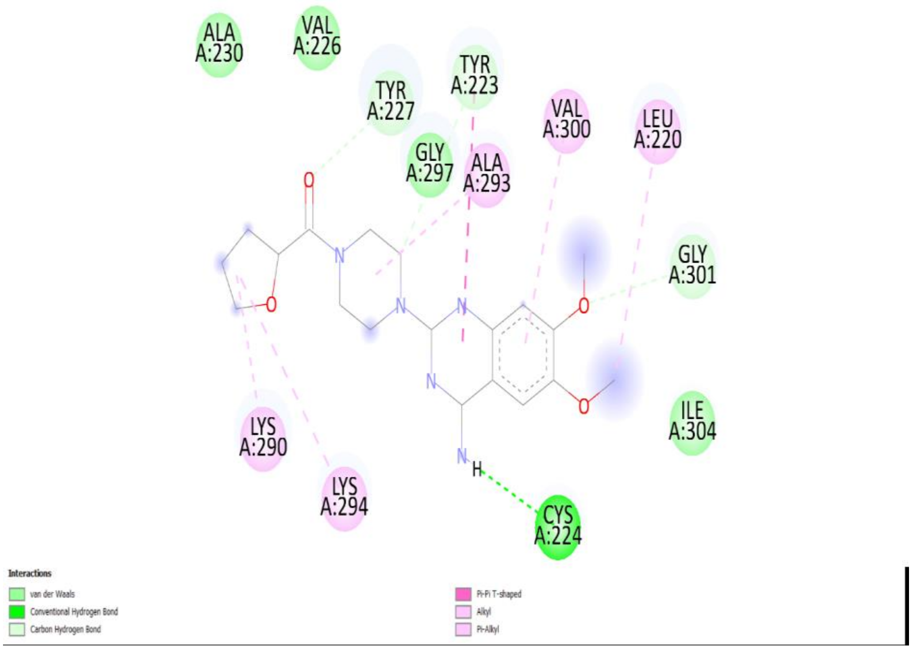


**1.i ABAR (human)+ Terazosin-HCL** [AlphaFold identifier of ABAR: AF-P35368-F1, SwissProt Accession No.: P35368]

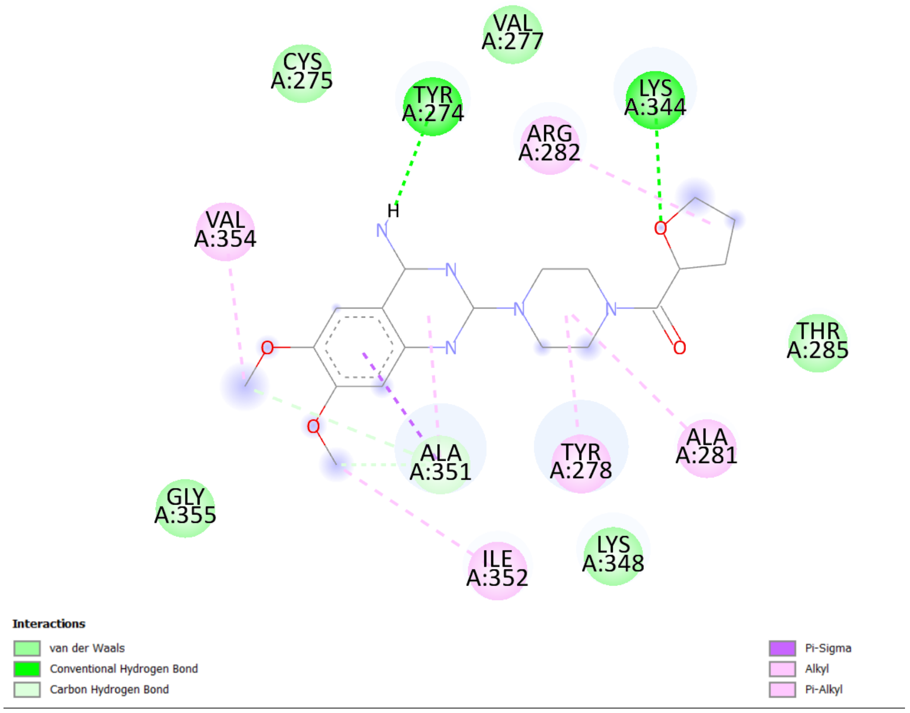


**1.j ADAR (human)+ Terazosin-HCL** [AlphaFold identifier of ADAR: AF-P25100-F1, SwissProt Accession No.: P25100]

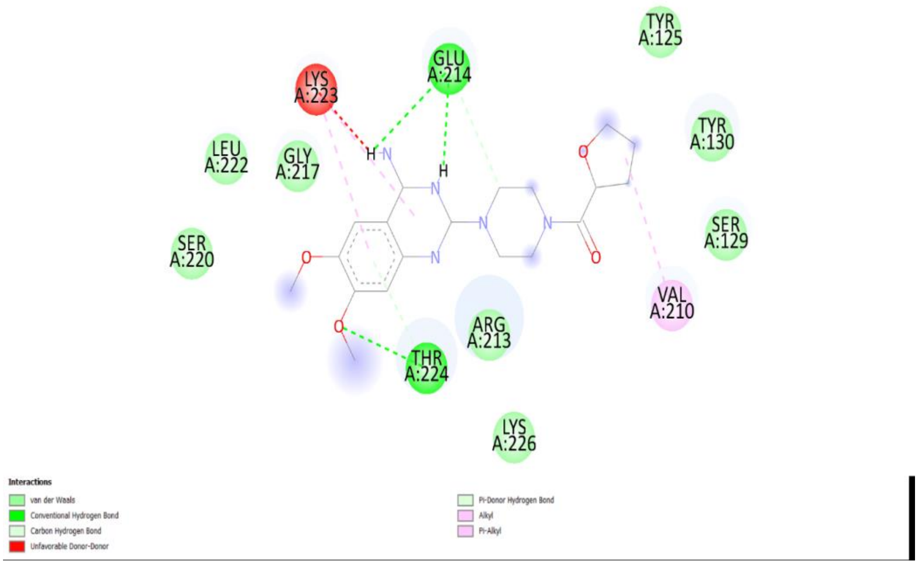


**1.k AAAR (Mouse)+ Terazosin-HCL** [AlphaFold identifier of AAAR: AF-P97718-F1, SwissProt Accession No.: P97718]

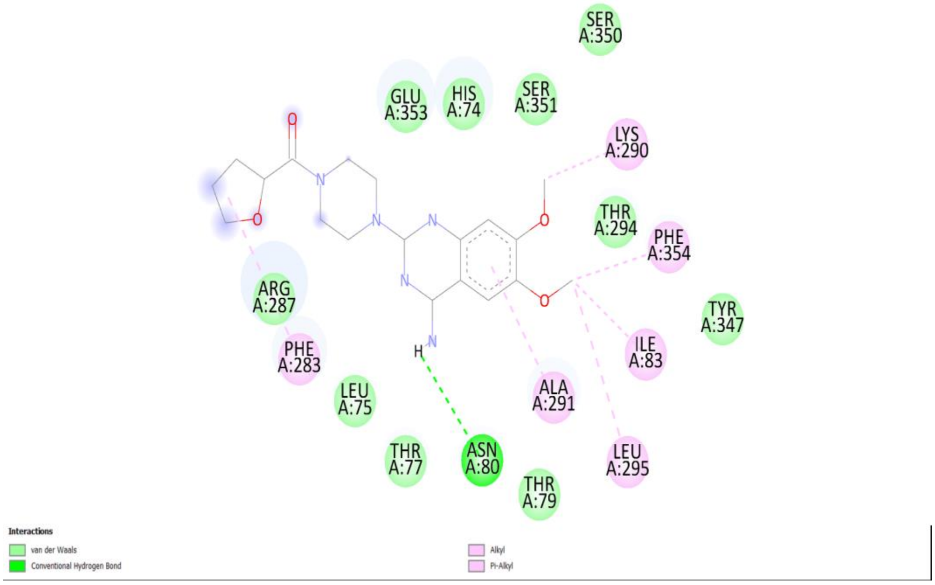


**1.l ABAR (Mouse)+ Terazosin-HCL** [AlphaFold identifier of ABAR: AF-P97717-F1, SwissProt Accession No.: P97717]

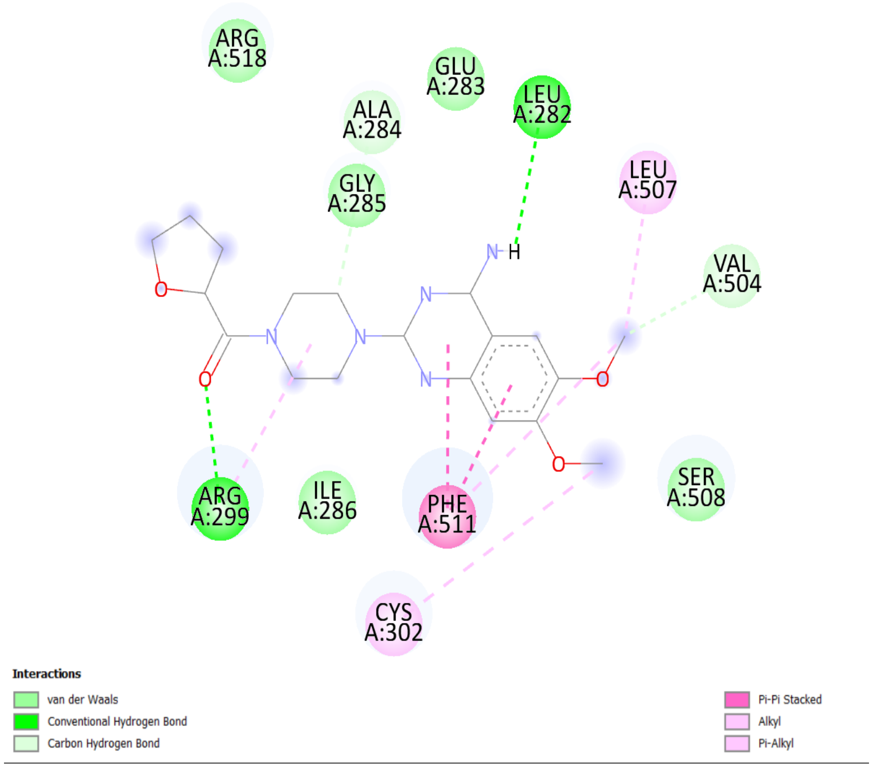


**1.m ADAR (Mouse)+ Terazosin-HCL** [AlphaFold identifier of ADAR: AF-P97714-F1, SwissProt Accession No.: P97714]

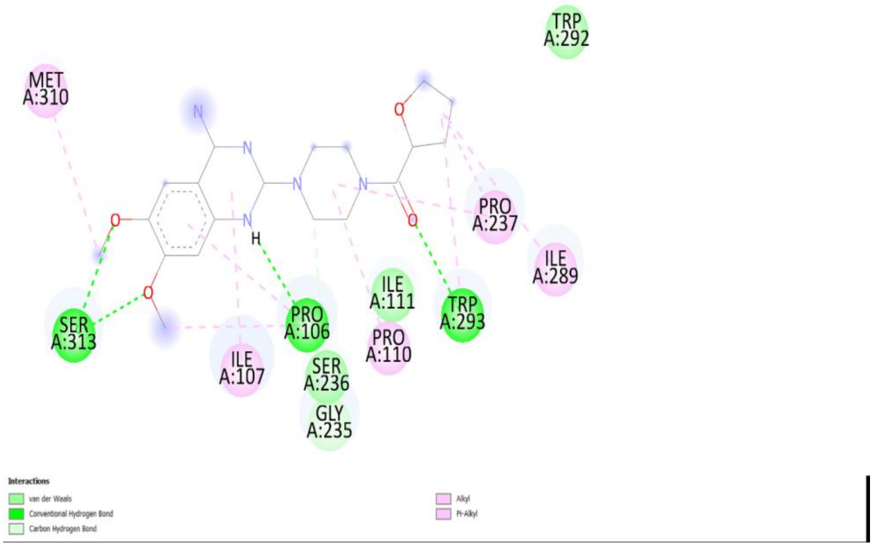


**1.n SER6 receptor (*C. elegans*)+ Terazosin-HCL** [AlphaFold identifier: AF-Q8MXS7-F1, SwissProt Accession No.: Q8MXS7]

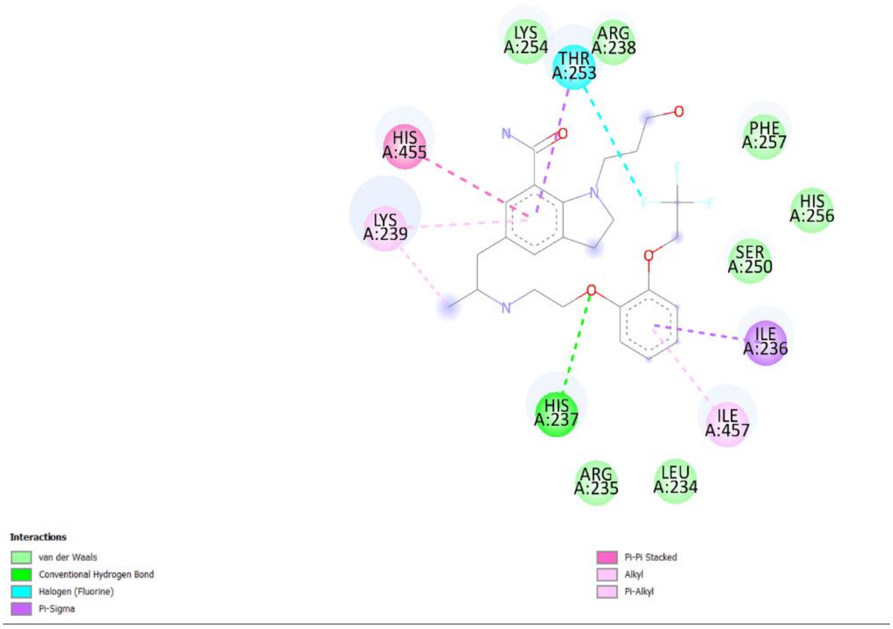


**1.o AAAR (human) + Silodosin** [AlphaFold identifier of AAAR: AF-P35348-F1, SwissProt Accession No.: P35348]

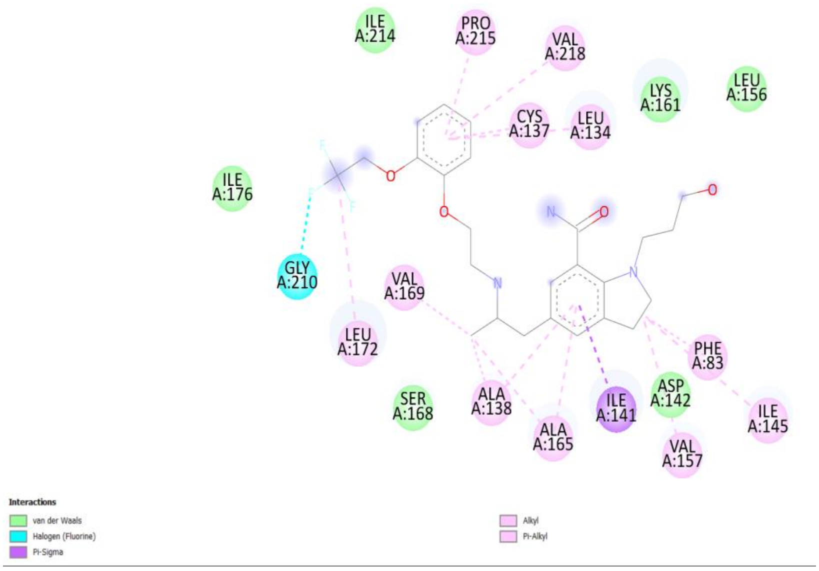


**1.p ABAR (human)+ Silodosin** [AlphaFold identifier of ABAR: AF-P35368-F1, SwissProt Accession No.: P35368]

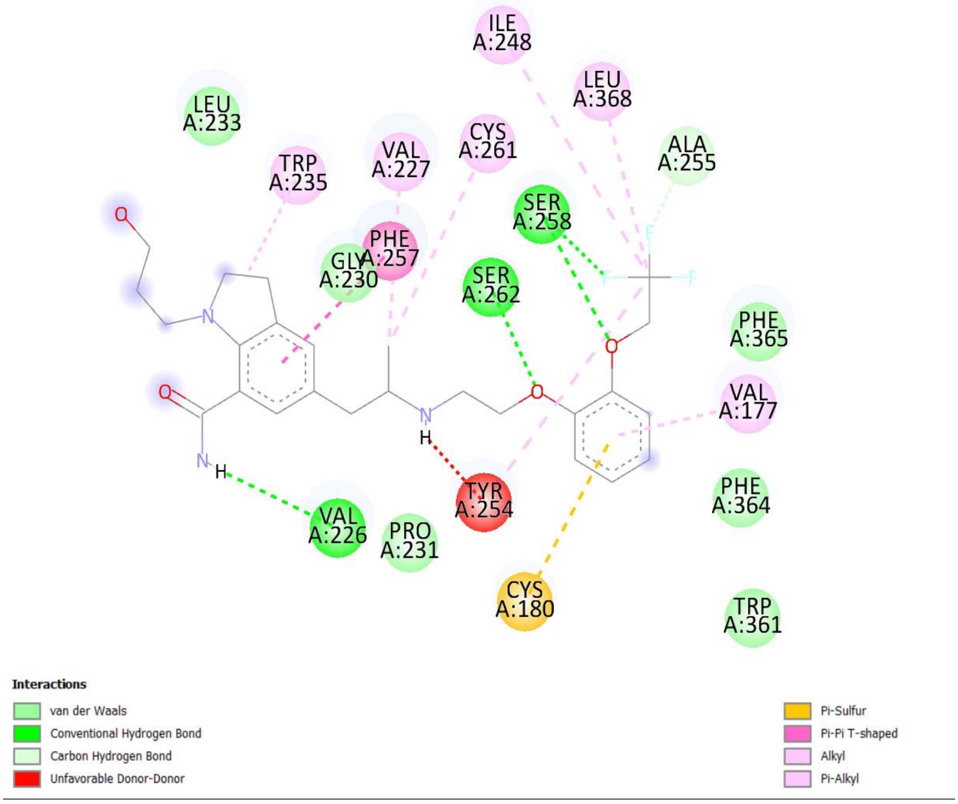


**1.q ADAR (human)+ Silodosin** [AlphaFold identifier of ADAR: AF-P25100-F1, SwissProt Accession No.: P25100]

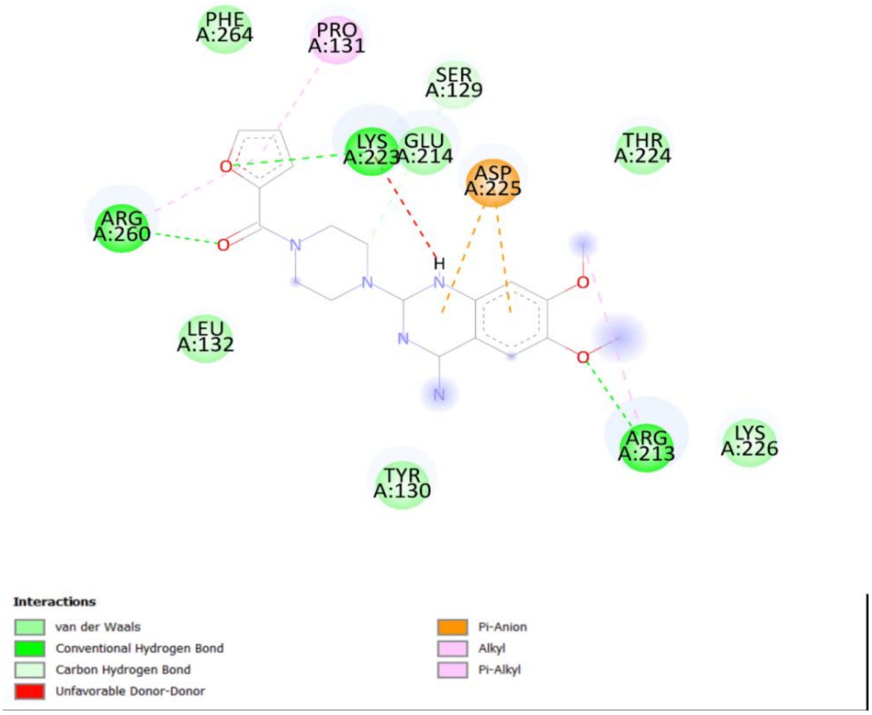


**1.r AAAR (Mouse)+ Silodosin**[AlphaFold identifier of AAAR: AF-P97718-F1, SwissProt Accession No.: P97718]

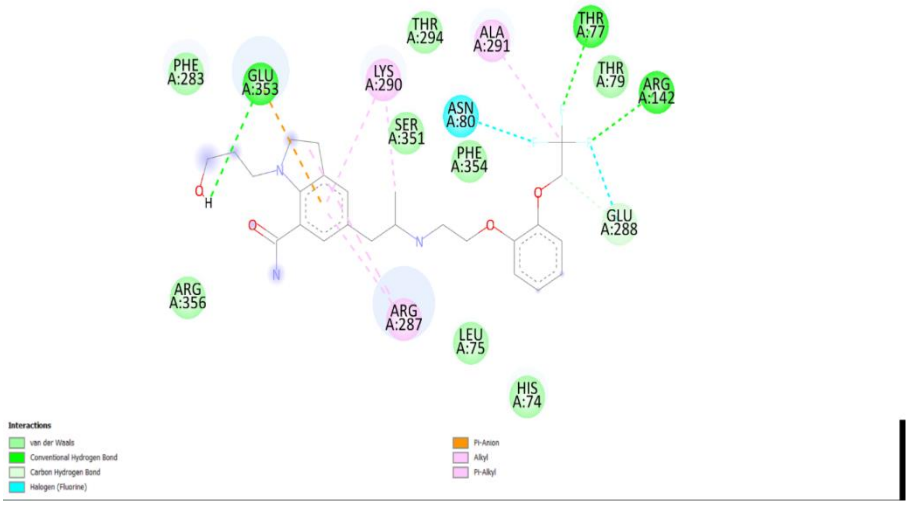


**1.s ABAR (Mouse)+ Silodosin** [AlphaFold identifier of ABAR: AF-P97717-F1, SwissProt Accession No.: P97717]

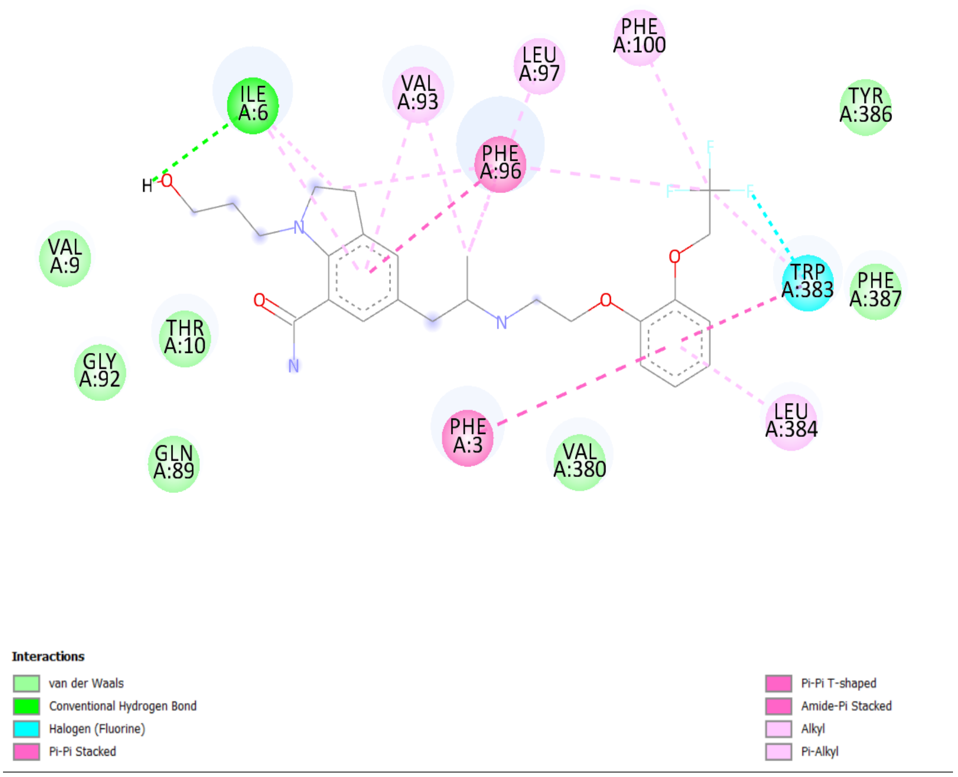


**1.t ADAR (Mouse)+ Silodosin** [AlphaFold identifier of ADAR: AF-P97714-F1, SwissProt Accession No.: P97714]

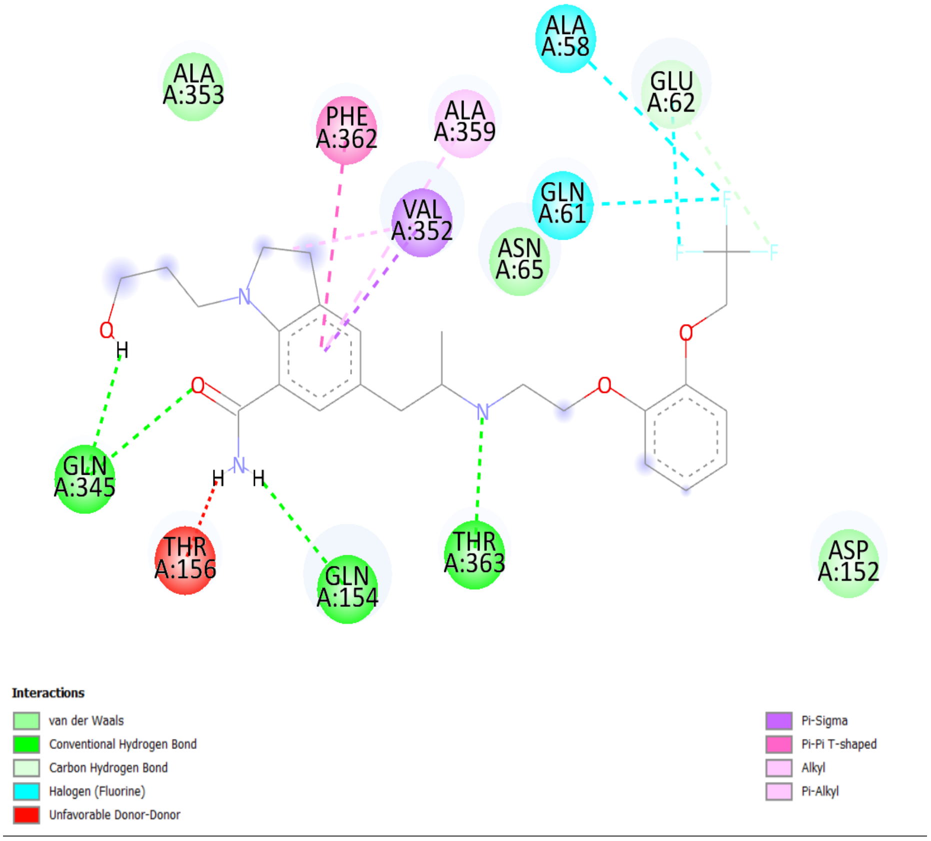


**1.u SER6 receptor (*C. elegans*)+ Silodosin** [AlphaFold identifier: AF-Q8MXS7-F1, SwissProt Accession No.: Q8MXS7]

**Supplementary Figure S1. a–u.**Homology modelled structures of the following proteins taken from SwissProt [Structure through AlphaFold]. Protein-ligand interactions of α1-adrenoreceptor antagonist (AAA) with α1-adrenergic receptor.

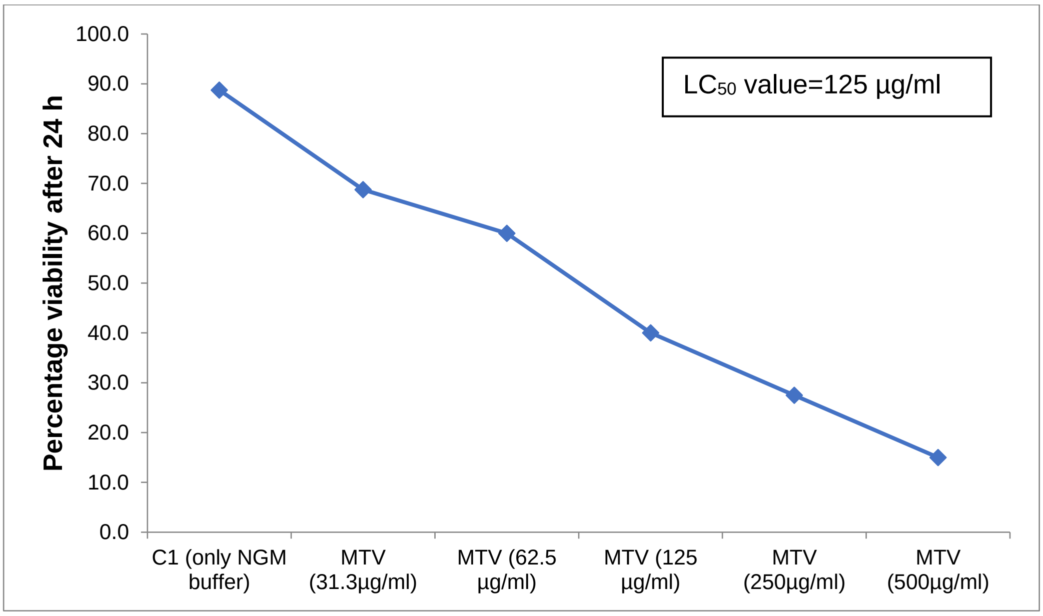


**Supplementary Figure S2.** The $LC_{50}$ value of *C. elegans* $N_2$ calculated after 24 h treatment of *M. tamulus* venom (MTV). The $LC_{50}$ value calculated for MTV towards *C. elegans*, after 24 h incubation, was 125 µg/ml.

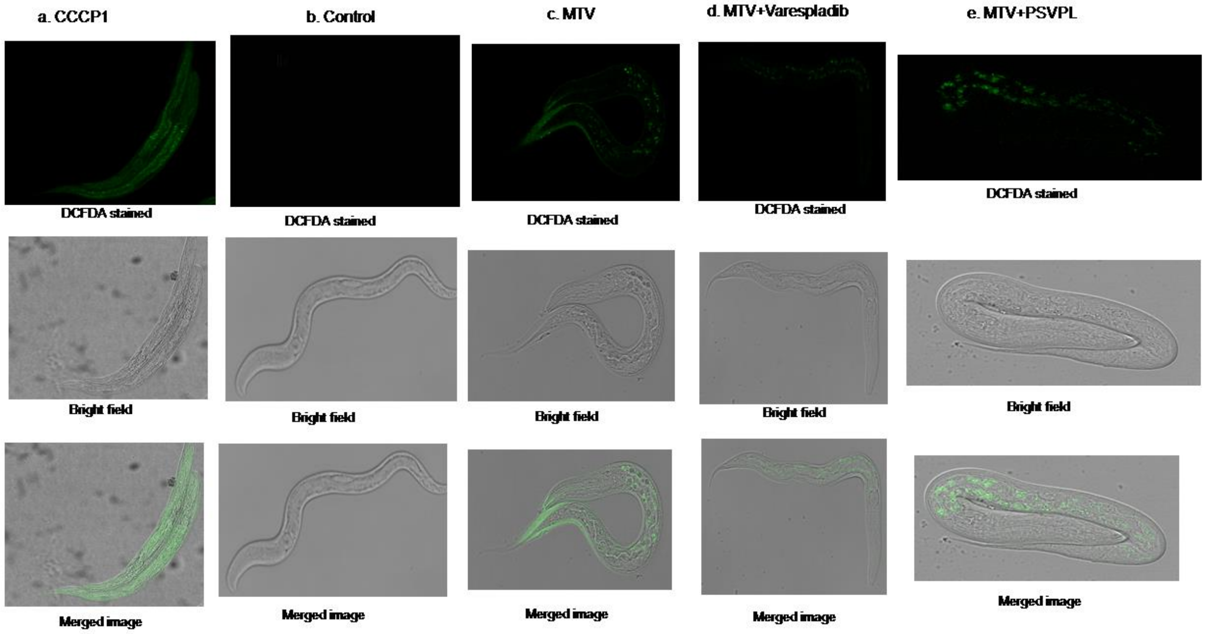

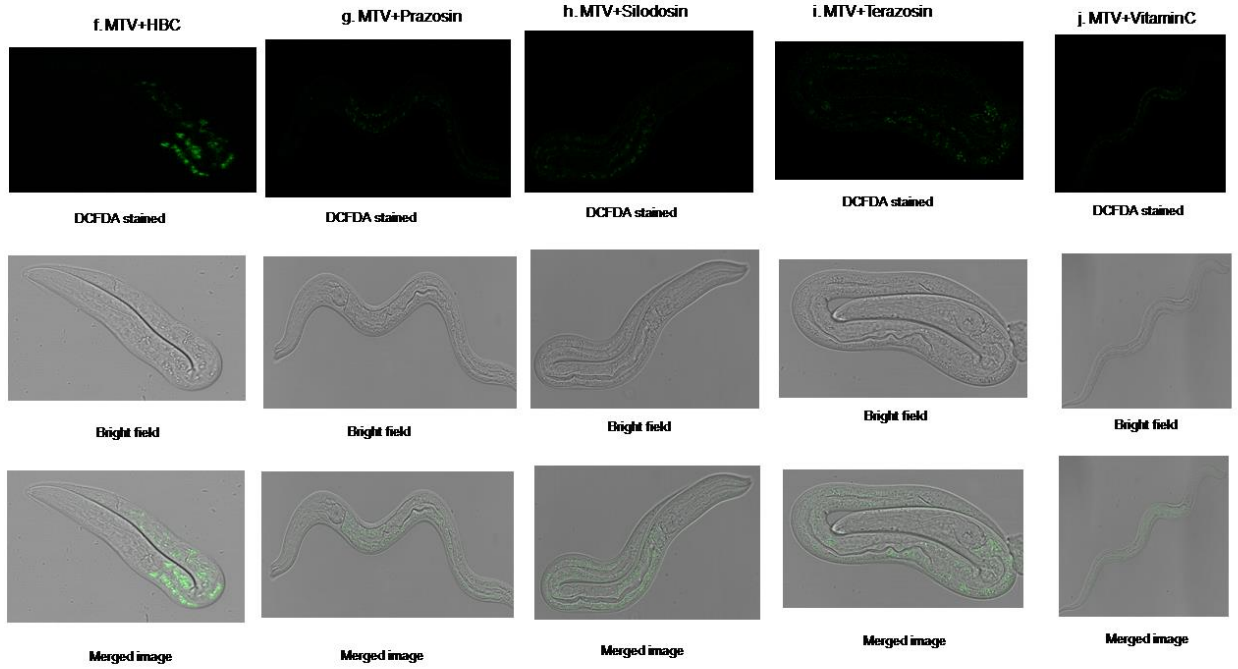


**Supplementary Figure S3.** Fluorescence image of confocal microscopy of *M. tamulus* venom induced ROS generation in *C. elegans* after 6h of *M. tamulus* venom ($LC_{50}$ concentration) treatment and its neutralization by Prazosin, Silodosin and Terazosin. ROS level in positive control (CCCP1) *C. elegans* was considered as baseline (100%) and other values were compared with that.

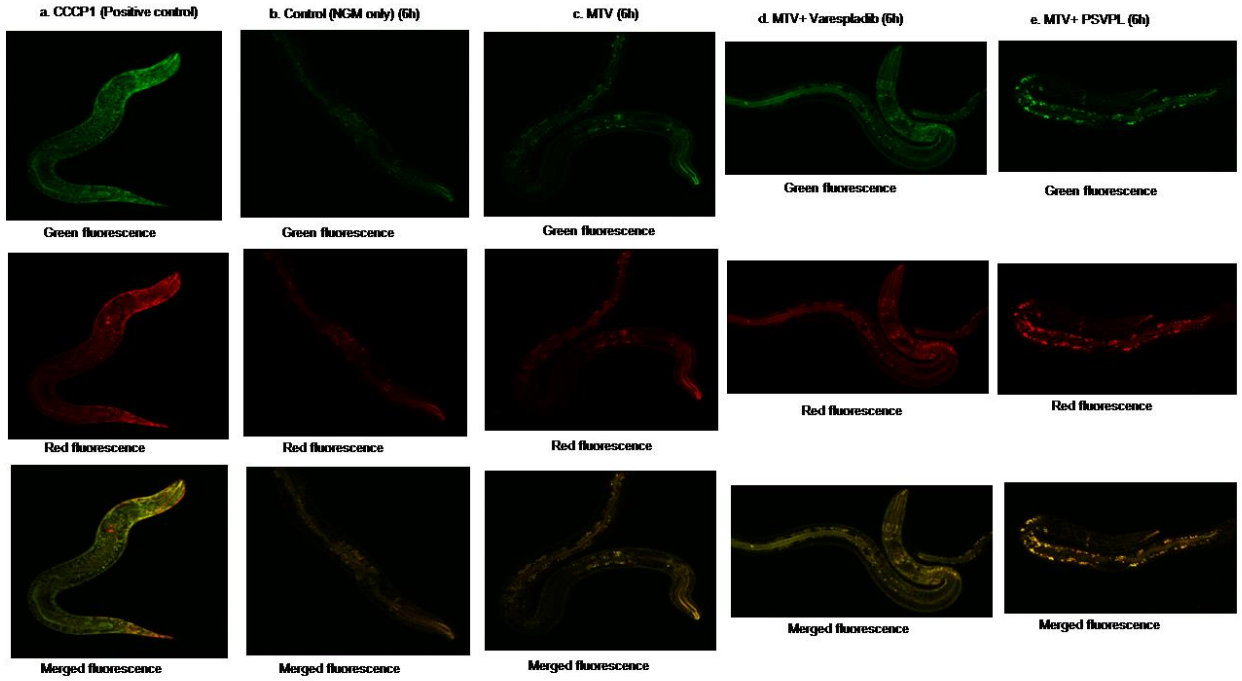

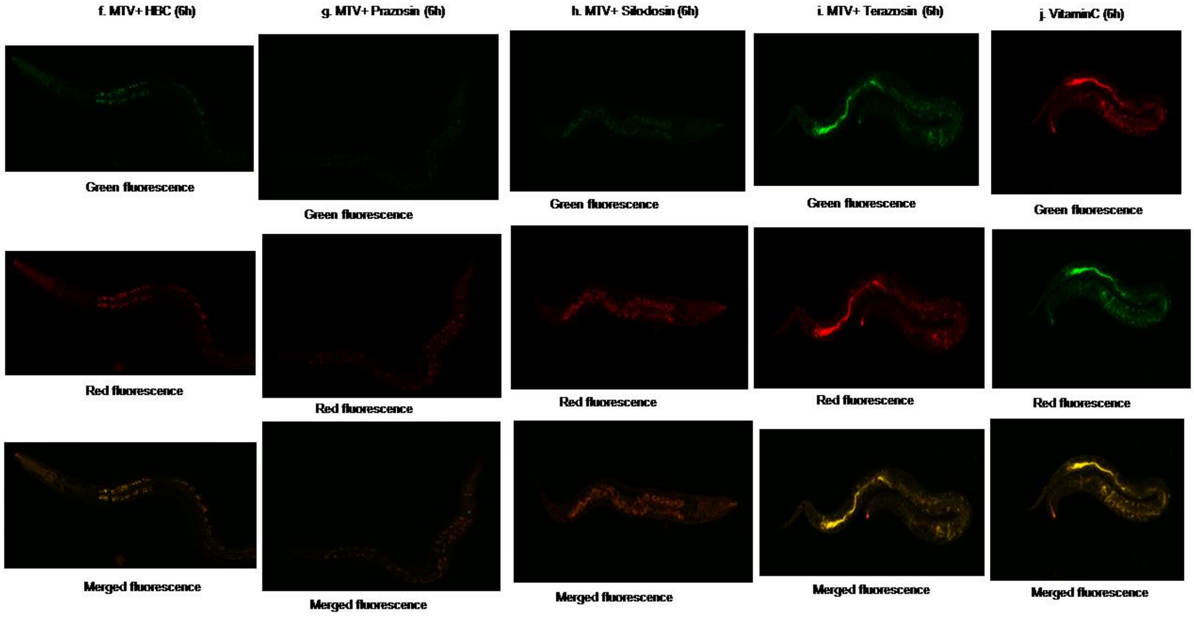
f. MTV+ HBC (6h)
g. MTV+ Prazosin (6h)
h. MTV+ Silodosin (6h)
i. MTV+ Terazosin (6h)
j. VitaminC (6h)
Green fluorescence
Green fluorescence
Green fluorescence
Green fluorescence
Green fluorescence
Red fluorescence
Red fluorescence
Red fluorescence
Red fluorescence
Red fluorescence
Merged fluorescence
Merged fluorescence
Merged fluorescence
Merged fluorescence
Merged fluorescence

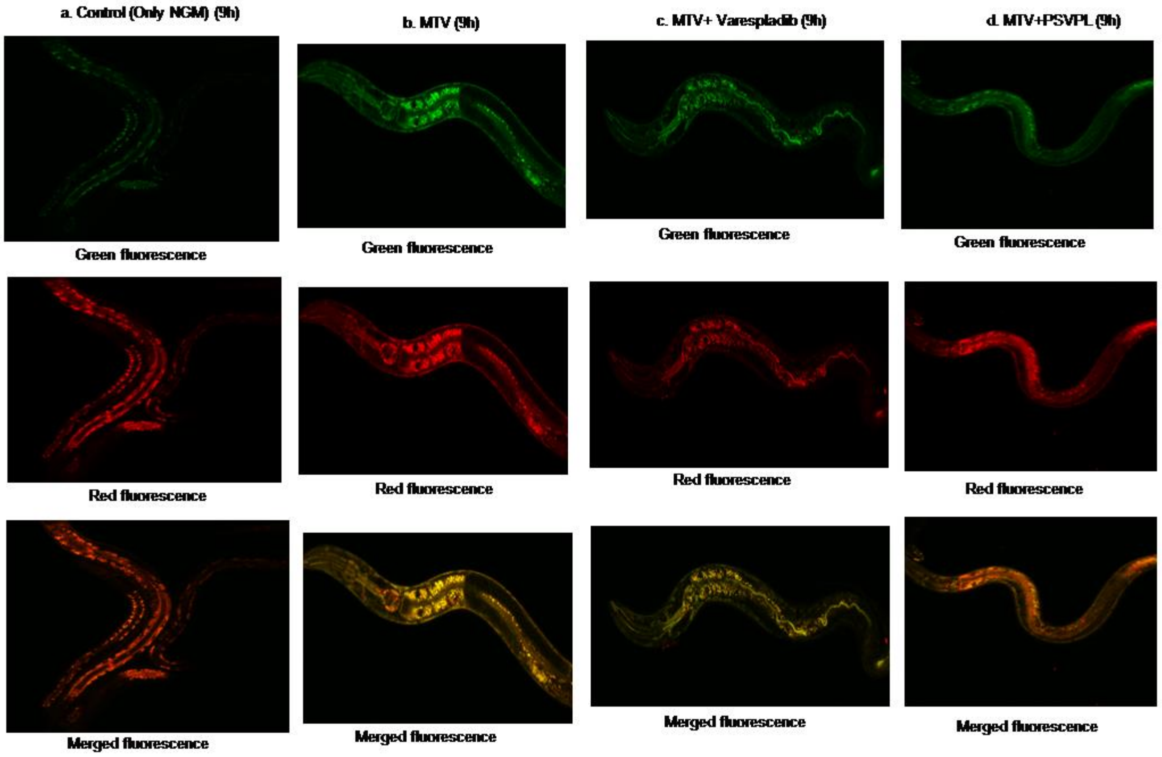
a. Control (Only NGM) (9h)
b. MTV (9h)
c. MTV+ Varespladib (9h)
d. MTV+PSVPL (9h)
Green fluorescence
Green fluorescence
Green fluorescence
Green fluorescence
Red fluorescence
Red fluorescence
Red fluorescence
Red fluorescence
Merged fluorescence
Merged fluorescence
Merged fluorescence
Merged fluorescence

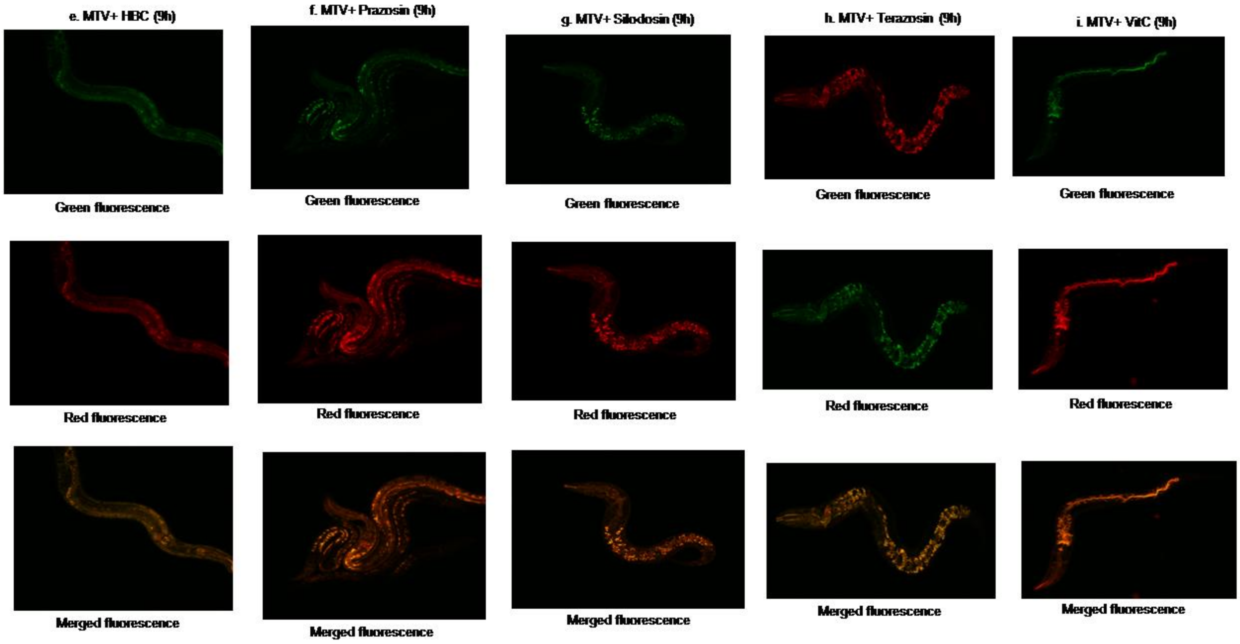
e. MTV+ HBC (9h)
f. MTV+ Prazosin (9h)
g. MTV+ Silodosin (9h)
h. MTV+ Terazosin (9h)
i. MTV+ VitC (9h)
Green fluorescence
Green fluorescence
Green fluorescence
Green fluorescence
Green fluorescence
Red fluorescence
Red fluorescence
Red fluorescence
Red fluorescence
Red fluorescence
Merged fluorescence
Merged fluorescence
Merged fluorescence
Merged fluorescence
Merged fluorescence

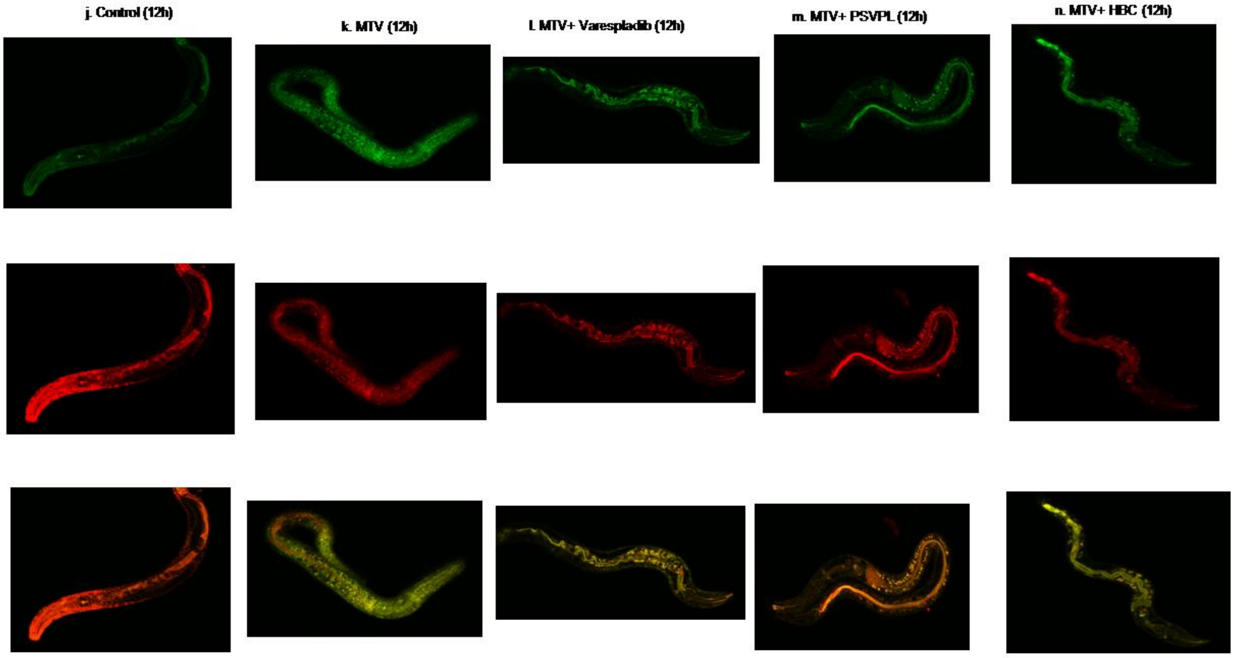
j. Control (12h)
k. MTV (12h)
l. MTV+ Varespladib (12h)
m. MTV+ PSVPL (12h)
n. MTV+ HBC (12h)

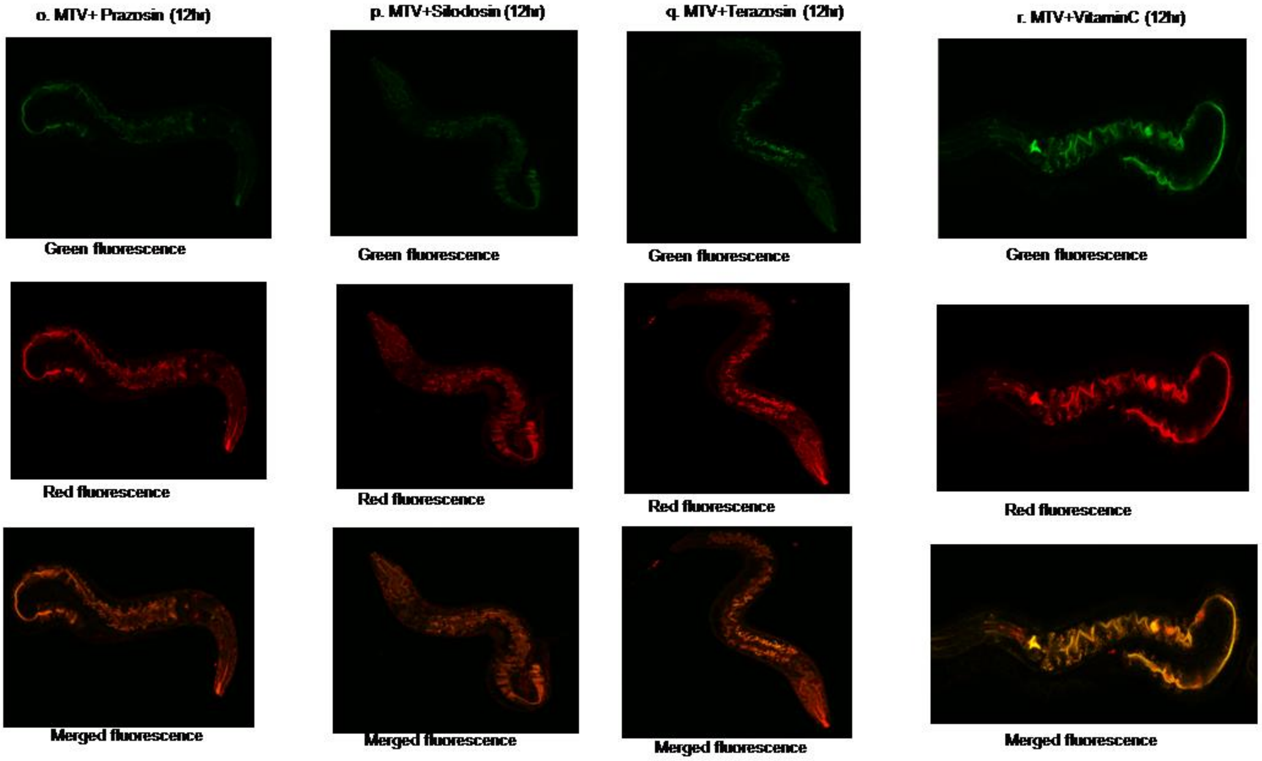


**Supplementary Figure S4.** Fluorescence image of confocal microscopy of MTV-induced alteration of mitochondrial membrane potential and its neutralization by Prazosin, Silodosin and Terazosin. ROS level in positive control (CCCP1) *C. elegans* was considered as baseline (100%) and other values were compared with that.

**a**

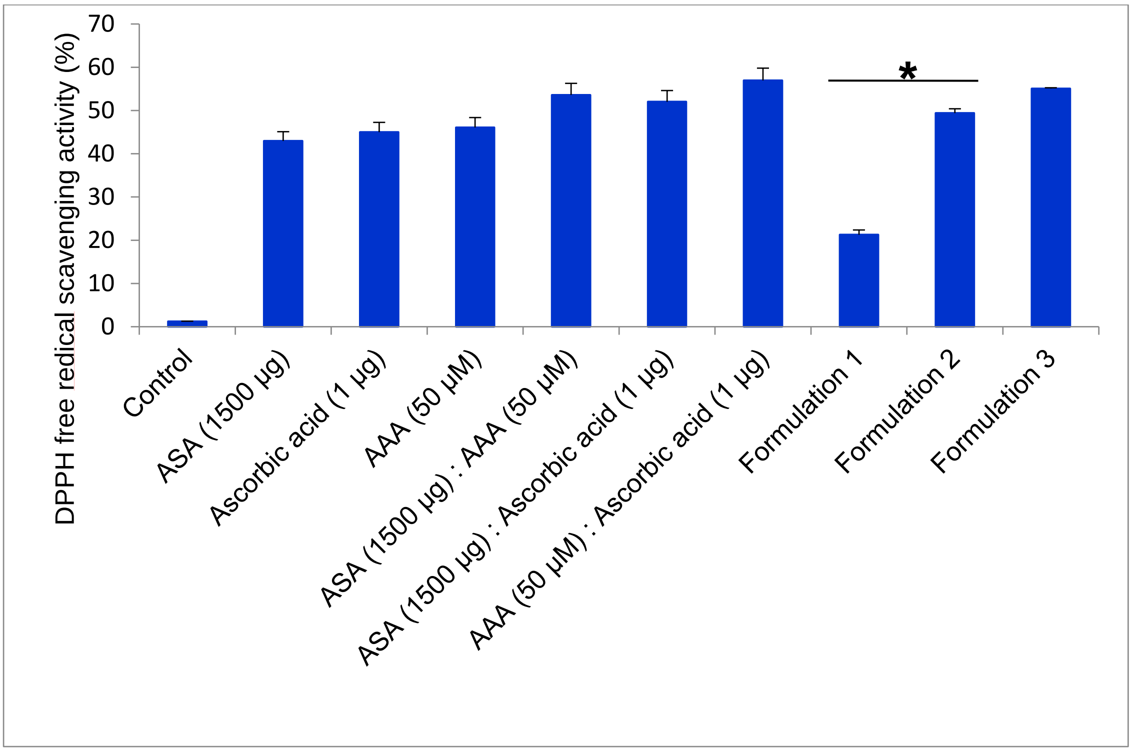


**b**

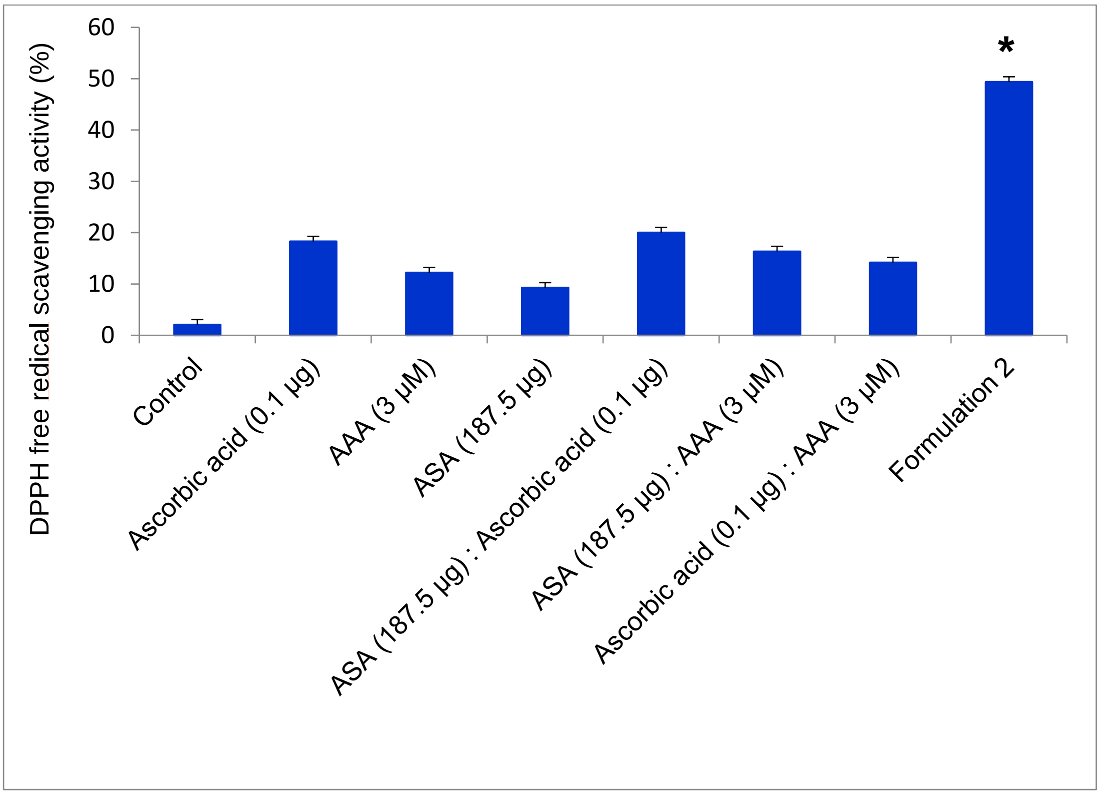


**Supplementary Figure S5.** shows the DPPH free radical-scavenging activity of **(a)** the optimum dose of individual formulation components, their combinations, and different concentrations of formulations, **(b)** Individual components of the formulation and their combinations compared with formulation 2. Data represent mean ± SD of three determinations. Significance of difference, *$p \leq 0.05$ as compared to formulation 2. There was no significant difference ($p > 0.05$) between formulations 2 and 3.

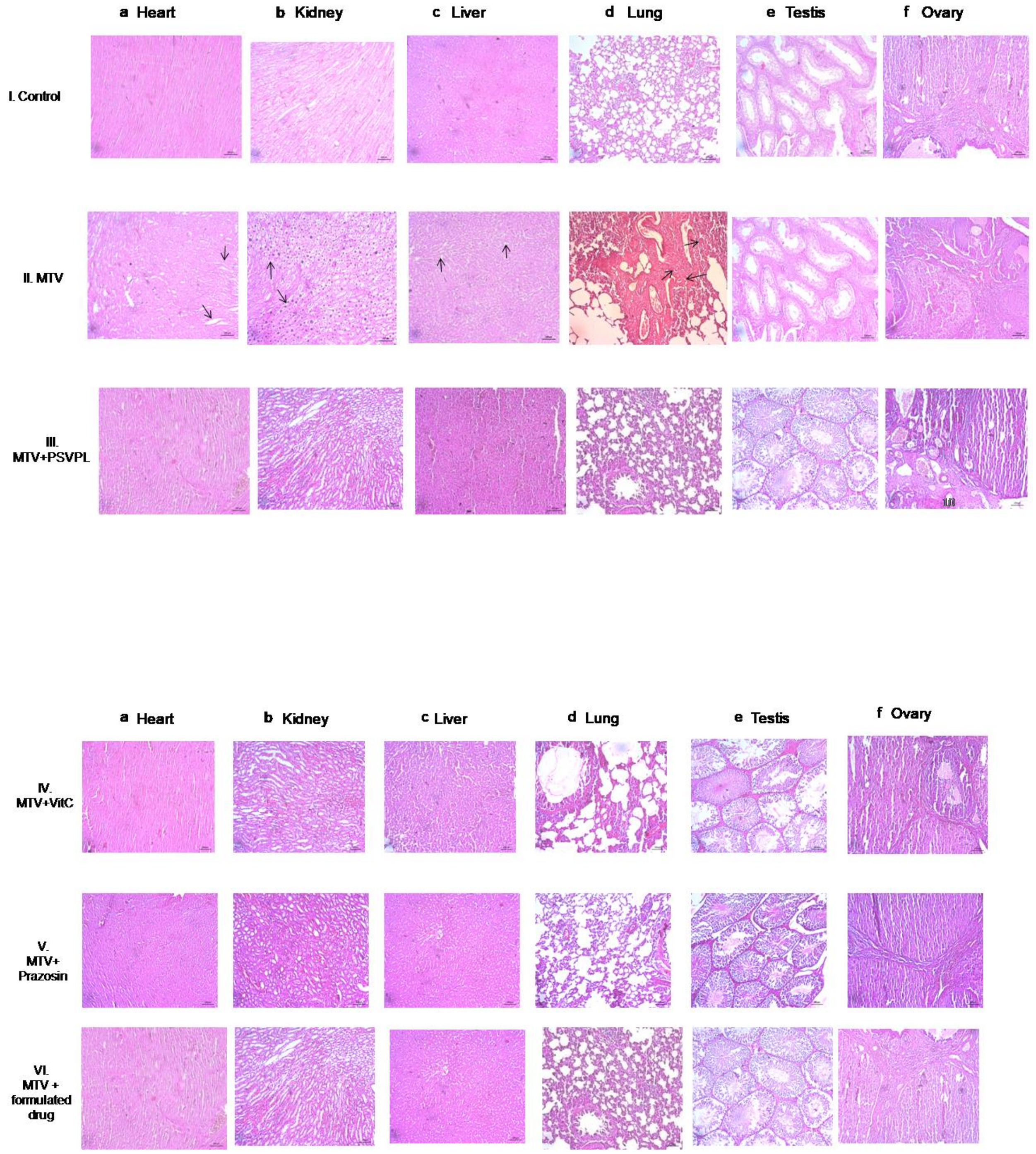


**Supplementary Figure S6.** Histopathological analysis of the *M. tamulus* venom-induced Wistar rat tissues and their neutralisation by formulation 2. Light microscopic observation of (a) Heart, (b) Kidney, (c) Liver, (d) Lung, (e) Testis and (f) Ovary from control and treated groups, Bar-100µM. The black arrow indicates the morphological changes observed in MTV-induced rat tissue compared to the control.

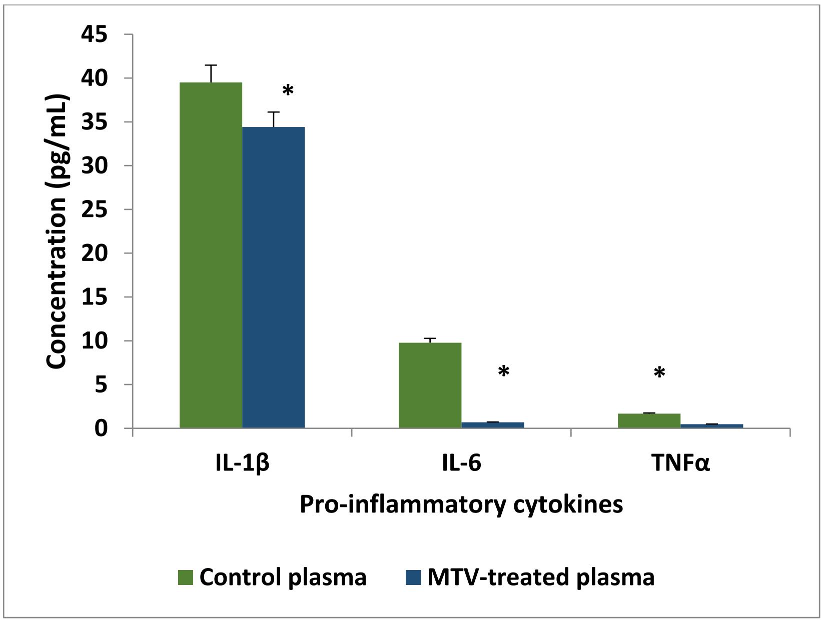


**Supplementary Figure S7.** Circulating levels of pro-inflammatory cytokines in the Swiss albino mice with and without *M. tamulus* envenomation, MTV-treated plasma and control plasma, respectively. Significance of difference, *$p \leq 0.05$ as compared to control
